# Dilute magnetism and edge-state engineering in monolayer SnO

**DOI:** 10.1039/d6na00140h

**Published:** 2026-06-02

**Authors:** Yuya Fukuta, Souren Adhikary, Kazuhito Tsukagoshi, Katsunori Wakabayashi

**Affiliations:** a Department of Nanotechnology for Sustainable Energy, School of Science and Technology, Kwansei Gakuin University Gakuen-Uegahara 1 Sanda 669-1330 Japan; b Research Center for Materials Nanoarchitectonics (MANA), National Institute for Materials Science (NIMS) Namiki 1-1 Tsukuba 305-0044 Japan WAKABAYASHI.Katsunori@nims.go.jp; c Center for Spintronics Research Network (CSRN), Osaka University Toyonaka 560-8531 Japan

## Abstract

Tin monoxide (SnO) is a p-type oxide semiconductor whose electronic properties can be widely modified *via* atomic-scale engineering. Using density functional theory, we investigate the electronic and magnetic properties of a transition-metal (TM = Mn, Fe, Co and W) doped SnO monolayer within a large supercell. We find that all dopants induce finite localized magnetic moments, primarily originating from d-orbitals of the impurity atoms. We show that these localized magnetic states give rise to nearly dispersionless bands in the vicinity of the Fermi energy (taking Co doped SnO as an example). In addition, we investigate dimensional effects by constructing nanoribbon geometries of the SnO monolayer. The ribbons exhibit intrinsic edge-localized states that are largely independent of ribbon width. For chiral nanoribbons oriented along a low-symmetry direction of the square lattice, we find that oxygen-rich edges are thermodynamically the most stable and remain semiconducting, whereas Sn-terminated edges host metallic one-dimensional conduction channels. Our results demonstrate that transition-metal doping and edge engineering provide effective routes to tailor the electronic properties of the SnO monolayer, making it a promising candidate for future spintronic and nanoelectronic applications.

## Abstract 2

Edge composition controls one-dimensional metallic channels in monolayer SnO nanoribbons.

## Graphical abstract

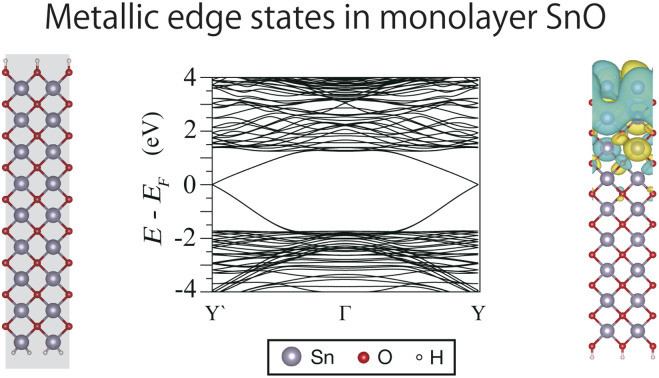

## 1. Introduction

Transparent and flexible electronics based on oxide semiconductors have been extensively investigated owing to their wide band gaps, chemical stability, and compatibility with low-temperature processing technologies, as summarized in several comprehensive reviews.^1–5^ While high-performance n-type oxide semiconductors are now well established, the realization of complementary transparent electronic circuits remains challenging due to the limited availability of p-type oxide semiconductors with sufficient carrier mobility and stability.

Among candidate p-type oxides, tin monoxide (SnO) is particularly attractive because the stereochemically active Sn^2+^ lone-pair states hybridize with O 2p orbitals, resulting in relatively dispersive valence-band states. Such lone-pair-driven band dispersion has been recognized as a key design principle for achieving p-type conductivity in oxide semiconductors.^3^ First-principles studies have further shown that monolayer SnO is dynamically stable and exhibits a moderate band gap together with high intrinsic hole mobility, making it a promising platform for two-dimensional (2D) oxide electronics.^6^ The electronic properties of SnO are further found to be highly sensitive to atomic-scale perturbations such as strain, native defects, and surface chemistry, which can introduce in-gap states and modify carrier transport.^7,8^

Beyond intrinsic band-structure considerations, carrier transport and stability in oxide semiconductors are known to be strongly influenced by oxygen-related defects and dopant chemistry. Experimental studies on In_2_O_3_-based amorphous oxide semiconductors have demonstrated that controlled suppression of oxygen vacancies and appropriate dopant incorporation are crucial for achieving stable and high-mobility transport characteristics in thin-film transistor devices.^9–11^ These experimental findings establish general design principles for oxide semiconductors that are also relevant to low-dimensional SnO systems. Recent experiments have demonstrated that tin monoxide can be stabilized in the atomically thin limit, retaining p-type conduction down to thicknesses of only a few atomic layers. This experimental realization establishes SnO as a viable 2D oxide platform, rather than a purely theoretical model system.^12^

A powerful strategy to further expand the functional landscape of SnO is transition-metal (TM) doping. Substitutional incorporation of 3d TM atoms has been predicted to introduce localized impurity states and magnetic moments, giving rise to spin-polarized electronic structures in the otherwise nonmagnetic SnO.^13–15^ In general, TM doping can induce dilute ferromagnetic or antiferromagnetic states in the host monolayer.^16,17^ In particular, cobalt doping has been proposed as a promising route to generate exchange-split Co-derived states near the Fermi level in SnO-based systems.^18,19^ Most previous theoretical studies have primarily relied on density-of-states analysis. However, electronic transport properties are strongly influenced by band dispersion. Therefore, in this work, we examine transition-metal doping in SnO monolayers by analyzing the band structure in addition to the density of states.

Reducing SnO to finite-width nanostructures introduces an additional design degree of freedom through edge formation.^20–22^ In a square-lattice system such as SnO, nanoribbons can be constructed along high-symmetry crystallographic directions as well as along low-symmetry (off-axis) directions with respect to the underlying lattice. Edges oriented along low-symmetry directions provide atomic configurations that are fundamentally distinct from high-symmetry terminations, leading to modified local coordination environments and potentially giving rise to unconventional edge-localized electronic states. Despite their importance, the electronic properties of such low-symmetry edges in SnO nanoribbons have not yet been systematically investigated.

In this work, we employ first-principles density functional theory calculations to systematically investigate the combined effects of transition-metal doping and edge engineering in monolayer SnO. We find that all TM dopants induce finite magnetic moments, primarily originating from localized d-orbitals of the impurity atoms. In particular, substitutional Co doping introduces strongly spin-polarized mid-gap states near the Fermi level. Notably, these mid-gap states exhibit nearly dispersionless (flat) bands in the vicinity of the Fermi level. The inclusion of on-site Coulomb interaction reveals a correlation-driven splitting of these flat bands, highlighting the importance of electron–electron interactions in accurately describing the electronic structure. Furthermore, we show that the dispersionless bands in Co-doped SnO lead to a reduced amplitude of the optical conductivity compared to the pristine SnO monolayer. In addition, we show that SnO nanoribbons exhibit intrinsic edge-localized states that are largely independent of ribbon width. Furthermore, nanoribbons with low-symmetry edge orientations exhibit a tunable transition between semiconducting and metallic behavior, depending on the atomic termination. These results establish atomistic design principles for controlling spin polarization, optical response, and edge-state conduction in two-dimensional SnO-based nanostructures.

## 2. Computational methods

First-principles calculations were performed within the framework of density functional theory (DFT) using the Vienna *ab initio* simulation package (VASP).^23,24^ The projector augmented-wave (PAW) method was employed to describe the interaction between valence electrons and ionic cores.^25,26^ Electron exchange and correlation were treated within the generalized gradient approximation (GGA) using the Perdew–Burke–Ernzerhof (PBE) functional.^27^ A plane-wave basis set with an energy cutoff of 500 eV was adopted, which was confirmed to be sufficient to ensure convergence of total energies and electronic structures. Brillouin-zone (BZ) integrations were carried out using Monkhorst–Pack *k*-point meshes.^28^ For 2D SnO monolayers, a *Γ*-centered mesh of 20 × 20 × 1 was employed, while denser one-dimensional *k*-point sampling was used along the periodic direction of SnO nanoribbons. A vacuum region of at least 15 Å was introduced to eliminate spurious interactions between periodically repeated images. All atomic structures were fully relaxed until the residual Hellmann–Feynman forces on each atom were less than 0.01 eV Å^−1^ and the total energy change between successive ionic steps was below 10^−6^ eV. Spin-polarized calculations were performed for transition-metal-doped systems. The electronic density of states and band structures were analyzed using the vaspkit package.^29^ On-site Coulomb interaction for the Co d orbitals was included within the DFT+U framework.^30^

To cross-check selected electronic and optical properties, additional calculations were performed using the quantum espresso (QE) package.^31,32^ Norm-conserving pseudopotentials were adopted as provided in the standard QE pseudopotential libraries.

The frequency-dependent optical properties were evaluated from the complex dielectric function *ε*(*ω*) = *ε*_1_(*ω*) + *iε*_2_(*ω*) obtained within the linear response formalism.^33–36^ The optical conductivity *σ*(*ω*) was calculated using the SI-unit relation

1 *σ*(*ω*) = *ε*_0_*ωε*_2_(*ω*),

where *ω* is the photon angular frequency and *ε*_0_ is the vacuum permittivity. Other optical quantities, including the refractive index *n*(*ω*), extinction coefficient *k*(*ω*), absorption coefficient *α*(*ω*), and energy-loss function *L*(*ω*), were derived from *ε*_1_(*ω*) and *ε*_2_(*ω*).

## 3. Results and discussion

### 3.1. Transition metal doping

**Fig. 1:**
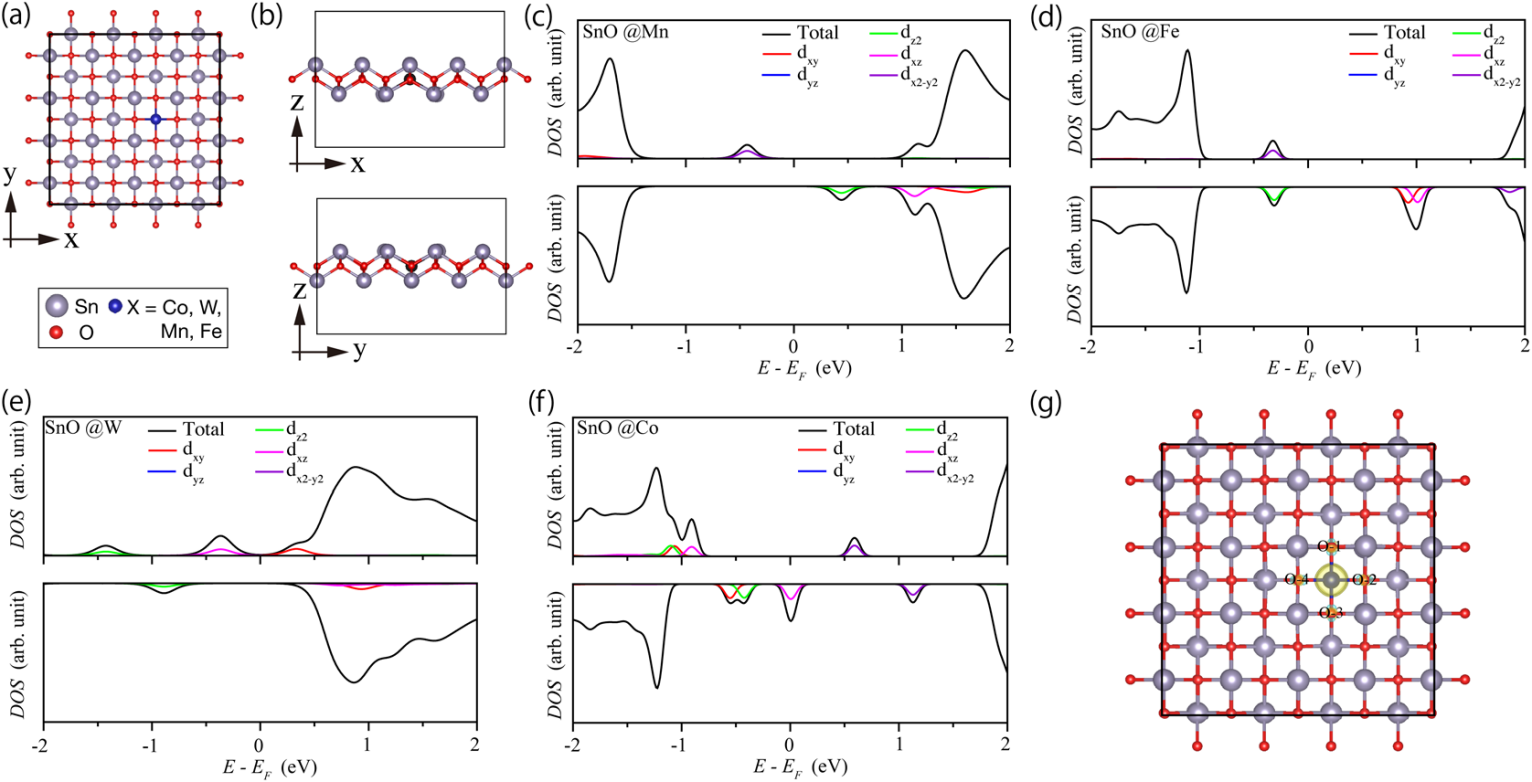
(a) and (b) Optimized top and side views of monolayer SnO structures doped with transition metals (TMs) in a 4 × 4 × 1 supercell, where one Sn atom is substituted by a TM atom (Mn, Fe, W, or Co). (c)–(f) Spin-resolved total density of states and projected density of states (PDOS) for Mn-, Fe-, W-, and Co-doped SnO, respectively. Different color lines are showing contributions from TM d orbitals. Among the considered dopants, Co doping exhibits the strongest spin asymmetry near the Fermi level (*E*_F_), with a spin-selective electronic structure. (g) Spin-density plot of the Co-doped SnO monolayer. The isosurface value is set to 0.001*e* Å^−3^.

Doping with transition-metal elements is a widely explored strategy for introducing spin polarization and magnetic functionality into semiconductors and oxides. In particular, dilute magnetic oxides have attracted sustained interest as potential building blocks for spintronic devices.^37^ In the case of monolayer SnO, substitutional incorporation of 3d TMs such as Co, Fe, Mn, or W provides an effective means to modify the electronic structure by introducing localized impurity states within the band gap and inducing spin polarization.^15,38–41^Fig. 1(a) and (b) present the top and side views, respectively, of the TM-doped SnO monolayer. In this work, we consider a 4 × 4 × 1 supercell of the SnO monolayer, where a TM atom substitutes a Sn atom (blue-colored atom). Fig. 1(c)–(f) show the total density of states (DOS) and projected density of states (PDOS) for Mn-, Fe-, W-, and Co-doped SnO monolayers, respectively, calculated using the DFT-PBE method. Since all doped systems become spin-polarized after TM substitution, the spin-up and spin-down DOS are plotted separately.

In all cases, impurity-derived states emerge near the Fermi level and exhibit pronounced spin asymmetry. The PDOS analysis reveals that these impurity states mainly originate from the d-orbitals of the dopant atoms. For Co-doped SnO, the PDOS indicates that the spin-up channel crosses the Fermi level, whereas the spin-down channel remains gapped, suggesting an apparent half-metallic electronic structure within the DFT-PBE approximation.^13,42^ This behavior originates from strong hybridization between Co 3d and O 2p orbitals, which gives rise to spin-polarized mid-gap states. In Fig. 1(g), we present the spin-density distribution of the Co-doped SnO monolayer (SnO@Co). The spin density is primarily localized around the Co atom and partially distributed over the neighboring O atoms, indicating hybridization between Co 3d and O 2p orbitals. Table 1 summarizes the magnetic moments of the dopant atoms and their neighboring O atoms in the TM-doped SnO monolayers. The magnitude of the magnetic moment strongly depends on the degree of hybridization between the dopant d-orbitals and the neighboring O atoms.

**Table 1:** Calculated local magnetic moment (in units of *µ*_B_) of the dopant atom and neighboring O atoms. The notations of O atoms are indicated in Fig. 1(g)

System	TM atom	O-1	O-2	O-3	O-4
SnO@Mn	4.300	0.031	0.031	0.031	0.031
SnO@Fe	3.498	0.064	0.064	0.064	0.064
SnO@W	1.524	0.019	0.019	0.019	0.019
SnO@Co	0.967	0.004	0.004	0.004	0.004

Thus, our PDOS analysis within the DFT-PBE approximation suggests that the Co-doped SnO monolayer exhibits half-metallic behavior. However, most previous theoretical studies have primarily focused on DOS/PDOS analysis, while the corresponding band dispersion has remained largely unexplored.^16–19^ Since the localized nature of impurity-induced states plays a crucial role in determining the electronic and magnetic properties of the system, we further investigate the electronic structure through spin-resolved band structure calculations.

**Fig. 2:**
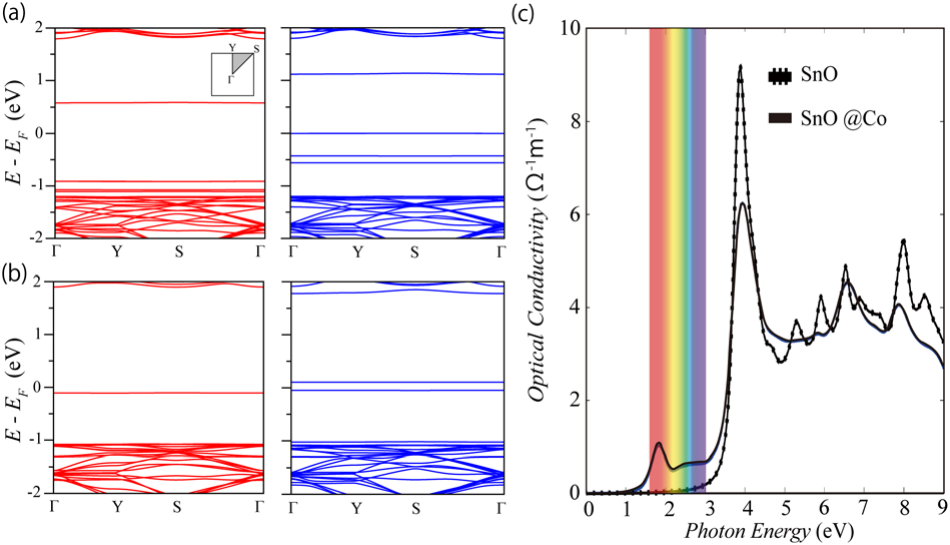
Electronic band structure of the Co doped SnO monolayer (a) without U (*i.e.*, *U* = 0 eV) and (b) with *U* = 3 eV. Red and blue bands represent up-spin and down-spin bands, respectively. The BZ is shown by the square. (c) Calculated optical conductivity spectra *σ*(*ω*) of pristine and Co-doped SnO, derived from the imaginary part of the dielectric function *ε*_2_(*ω*) according to eqn (1).

As a representative example, we focus on the Co-doped SnO monolayer (SnO@Co), which shows apparent half-metallicity within DFT-PBE. Fig. 2(a) presents the spin-polarized electronic band structure of SnO@Co, where the red and blue bands correspond to the spin-up and spin-down channels, respectively. Notably, the bands appearing near the Fermi level are nearly dispersionless (flat) throughout the BZ. The absence of significant band dispersion indicates strong localization of the impurity-induced states around the Co atom, consistent with the spin-density distribution shown in Fig. 1(g). Consequently, charge transport associated with these states is expected to be negligible due to their vanishing group velocity.

To further examine correlation effects, we performed DFT+U calculations and present the corresponding band structure for *U* = 3 eV in Fig. 2(b) (results for other *U* values are provided in the SI, see Fig. S1). The inclusion of on-site Coulomb interaction leads to a correlation-driven splitting of the flat bands near the Fermi level, resulting in the destruction of the half-metallic character predicted by standard DFT-PBE. Note that we also varied the on-site Coulomb interaction parameter *U* from 1 to 2 eV (see Fig. S1). In both cases, the half-metallic character disappears; however, the overall nature of the band structure remains qualitatively similar to that obtained for *U* = 3 eV. These results demonstrate that the magnetic impurity states are highly localized and non-itinerant in nature, implying negligible electronic transport through these states.

The non-itinerant nature of the electronic states near the Fermi level is further confirmed by the optical conductivity of the Co-doped SnO system. Fig. 2(c) shows the calculated optical conductivity, *σ*(*ω*), for pristine and Co-doped SnO monolayers. A clear redshift of the absorption edge is observed in the Co-doped system, indicating the onset of optical absorption at lower photon energies due to the impurity-induced states near the Fermi level.^1,43,44^ However, the magnitude of the optical conductivity is reduced compared to that of the pristine SnO monolayer. This suppressed optical response originates from the highly localized and nearly dispersionless nature of the Co-induced electronic states, which possess negligible carrier mobility. Overall, our results demonstrate that Co doping in SnO monolayers induces dilute magnetism accompanied by non-itinerant electronic states, highlighting the importance of localization and electron correlation in determining the electronic and optical properties of the system.

### 3.2. Edge states in SnO nanoribbons

From a technological viewpoint, it is noteworthy that state-of-the-art silicon devices have already achieved effective channel widths of approximately 10 nm and are rapidly approaching the single-nanometer scale. At such dimensions, edge and boundary effects inevitably play a central role in determining electronic transport, rendering nanoribbon models particularly relevant for exploring intrinsic low-dimensional physics.^45^

**Fig. 3:**
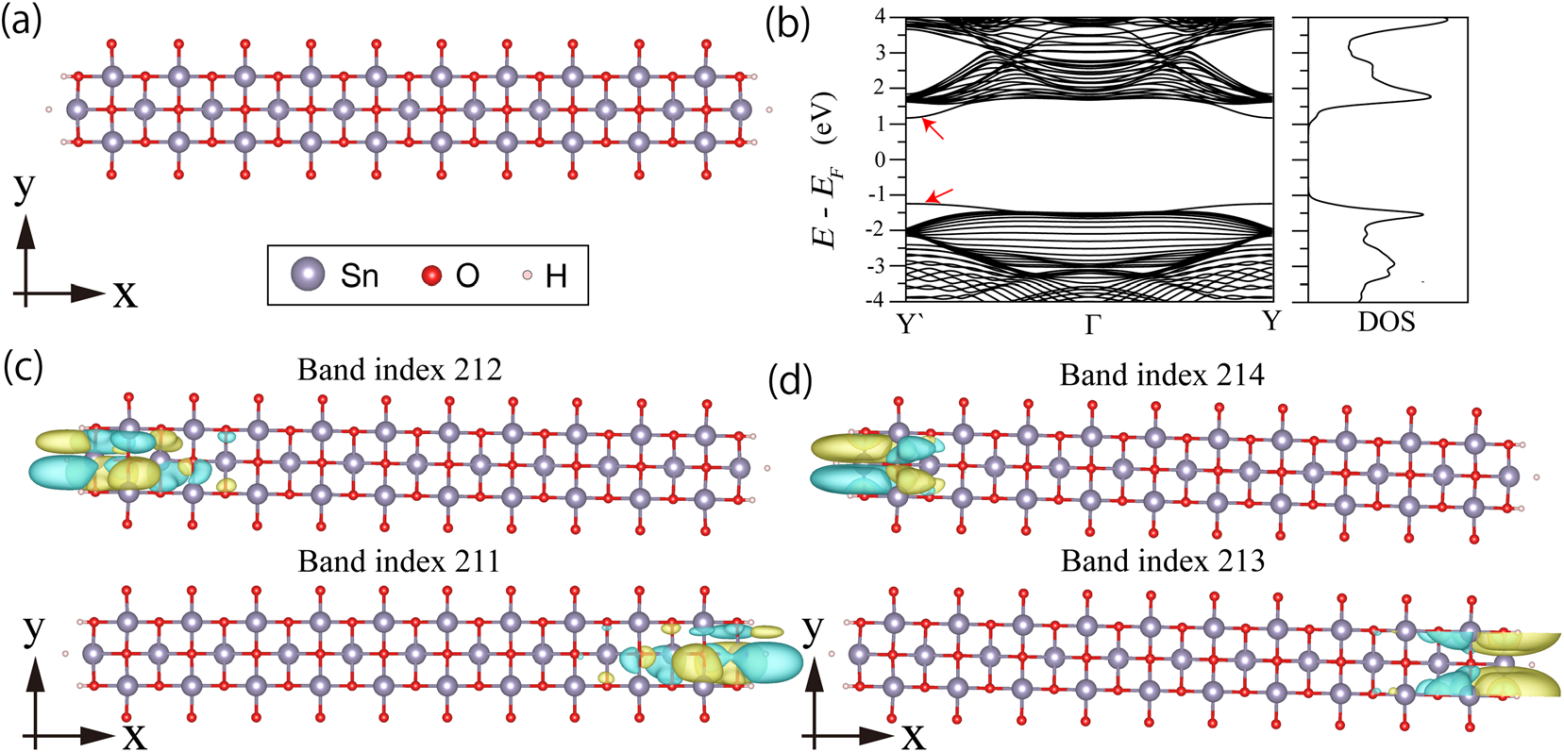
(a) Atomic structure of a hydrogen-terminated SnO nanoribbon, where Sn, O, and H atoms are represented by purple, red, and white spheres, respectively. (b) Calculated electronic band structure and density of states (DOS), showing additional in-gap bands (shown by the red arrows) near the Fermi level (*E*_F_) that are absent in the pristine monolayer. (c) and (d) Real-space isosurfaces of the wavefunctions corresponding to the in-gap bands (band indices 211–212 are degenerate valence bands and 213–214 are degenerate conduction bands). The charge density is strongly localized at the ribbon edges, consistent with edge-derived electronic states. Each in-gap band is doubly degenerate, reflecting the presence of two structurally equivalent edges in the nanoribbon. The isosurface value is set to 0.001*e* Å^−3^.

To examine how edge structures influence the electronic properties of SnO, we constructed hydrogen-terminated SnO nanoribbons and carried out first-principles DFT calculations. While edge-induced electronic states are well established in low-dimensional lattice systems, as originally revealed in early theoretical studies and later elaborated by first-principles calculations,^46,47^ their manifestation in square-lattice oxide nanoribbons such as SnO remains largely unexplored. Fig. 3(a) displays the atomic geometry, electronic band structure, and wavefunction distributions for a representative SnO nanoribbon. Although hydrogen passivation is introduced primarily to eliminate dangling-bond artifacts, the edge-localized states persist even after passivation. Their energetic positions and spatial distributions are modified depending on the specific edge chemistry, indicating that these states are intrinsic features of the ribbon geometry rather than passivation-induced states.^47^

To evaluate the relative stability of different edge structures, we considered three passivation schemes: (i) without hydrogen termination, (ii) hydrogen bonded only to Sn atoms, and (iii) hydrogen bonded to both Sn and O atoms. The corresponding relative total energies were calculated using QE and are summarized in Table 2. Such energetic comparisons are commonly employed to evaluate the stability of low-dimensional edge-terminated nanostructures.^48^ Among the three configurations, the fully hydrogen-passivated Sn–H–O edge is the most stable, being lower in energy by 34.54 eV per supercell than the unpassivated edge. The Sn–H terminated configuration is also stabilized by 16.26 eV per supercell. These results indicate that complete hydrogen passivation is energetically favorable and provides a realistic structural model for investigating the intrinsic edge states of SnO nanoribbons.

**Table 2:** Relative total energies of hydrogen-terminated SnO nanoribbons with different edge configurations. Energies are given per supercell and referenced to the unpassivated ribbon. The values are rounded to two decimal places

Termination	Relative energy (eV)
Without H	0.00
Sn–H	−16.26
Sn–H–O	−34.54

In Fig. 3(b), we present the band structure and DOS of SnO nanoribbons. From the band structure, additional bands (marked by the red arrow) appear in the vicinity of the Fermi level, which are absent in the pristine monolayer. The corresponding wavefunction isosurfaces in Fig. 3(c) and (d) demonstrate that these states are strongly confined to the Sn- and O-terminated ribbon edges. Each in-gap band is doubly degenerate (see the band index), reflecting the presence of two equivalent edges. Such strong localization suggests that the edge states can function as one-dimensional conductive channels running along the ribbon boundaries.^9,10^ Furthermore, we confirm that these edge states are largely independent of the ribbon width. To verify this behavior, we investigated width-dependent electronic structures of SnO nanoribbons with both larger and smaller widths compared to the ribbon shown in Fig. 3(a). The corresponding results are presented in Fig. S2 in the SI. In all cases, edge-localized states persist, while only the band gap changes with ribbon width. These results demonstrate that the emergence of edge states is an intrinsic feature of the SnO nanoribbon geometry and is robust against variations in ribbon width.

Importantly, the edge states appear for all edge termination types, demonstrating that they are intrinsic electronic features of SnO nanoribbons rather than artifacts of surface chemistry. Such sensitivity to local coordination and defect chemistry is a characteristic feature of oxide semiconductors in general. In experimental oxide systems, including In_2_O_3_-based thin-film transistors, transport properties have been shown to depend critically on oxygen vacancy concentration and local bonding environments.^9,11^ Their energetic tunability through edge passivation indicates that the electronic structure of the ribbon boundaries can be systematically controlled, providing a practical route for engineering edge-dominated conduction channels in low-dimensional oxide nanostructures.^49^

### 3.3. Chiral edge effects

**Fig. 4:**
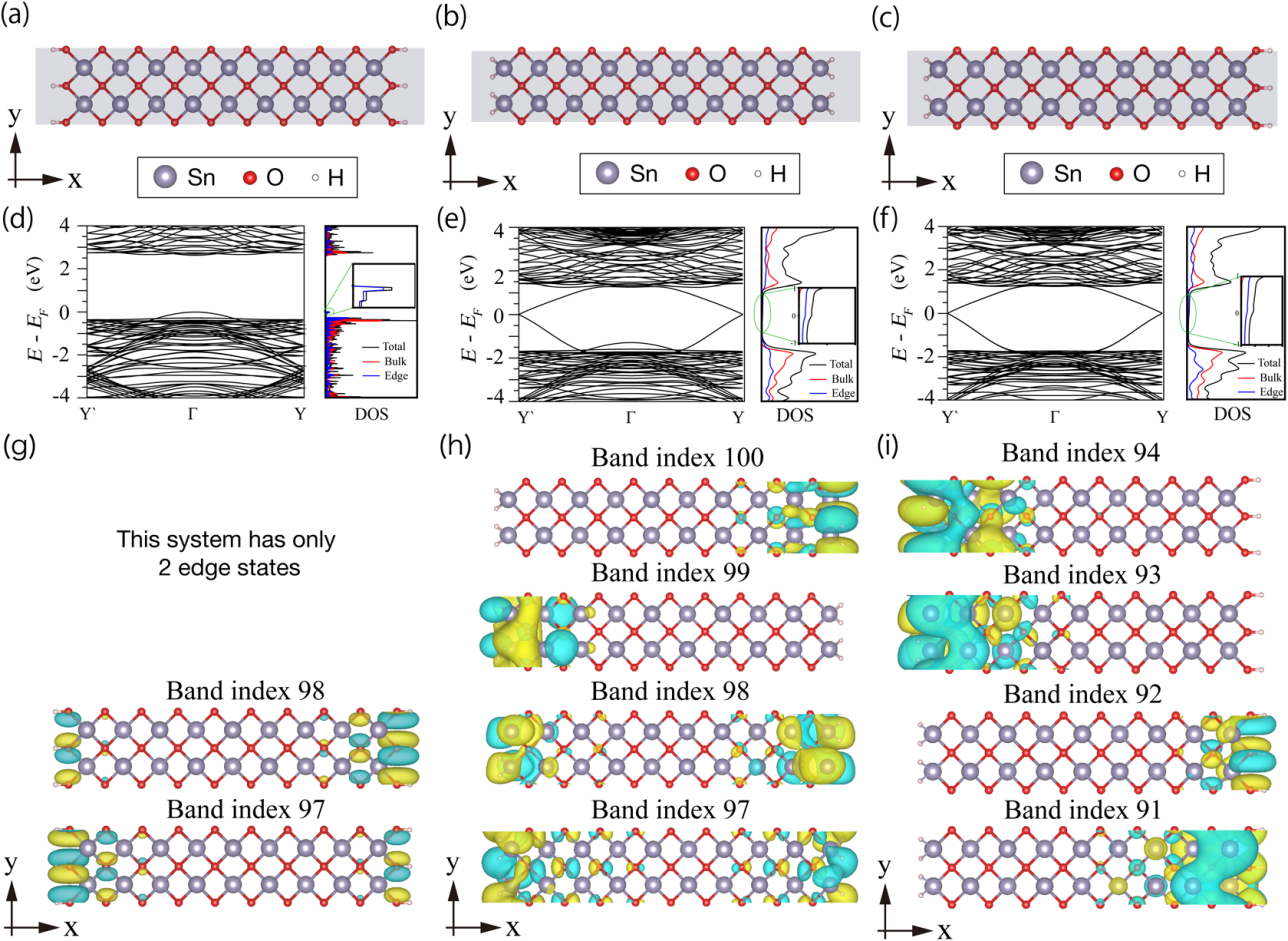
(a)–(c) Atomic structures of hydrogen-passivated SnO nanoribbons oriented along a low-symmetry (45°) direction with respect to the square lattice. The three edge terminations considered are (a) O–O, (b) Sn–Sn, and (c) Sn–O, which give rise to chemically and structurally inequivalent ribbon edges. (d)–(f) Corresponding electronic band structures and densities of states (DOS). For the O–O terminated ribbon, edge-derived states remain localized within the band gap without crossing the Fermi level, preserving semiconducting behavior. In contrast, the Sn–Sn and Sn–O terminated ribbons exhibit metallic edge states that intersect the Fermi level, indicating the formation of one-dimensional conducting channels. In DOS plots, the red line and blue line show bulk and edge atoms contributions. The zoomed-in view is shown in the inset. (g)–(i) Real-space wavefunction isosurfaces shown below each panel confirm strong localization of these states at the ribbon edges. The wavefunctions are evaluated at *k*-points located at the midpoint between *Γ* and *Y* for all three ribbon configurations. The two colors represent opposite phases of the wavefunction, with yellow and cyan corresponding to positive and negative signs, respectively.

To further clarify how edge geometry influences the electronic properties of SnO, we constructed nanoribbons oriented along a low-symmetry (45°) direction with respect to the underlying square lattice. This chiral orientation corresponds to a cutting direction that is not aligned with the principal crystallographic axes, leading to atomic edge configurations that are not related by mirror or rotational symmetry. As a result, the two ribbon edges become chemically and structurally inequivalent, giving rise to distinct electronic environments. As shown in Fig. 4(a)–(c), three representative edge terminations were examined: (i) oxygen-terminated edges (O–O), (ii) tin-terminated edges (Sn–Sn), and (iii) mixed tin–oxygen termination (Sn–O). All structures were hydrogen-passivated using the most stable scheme identified previously.

DFT calculations reveal that all three chiral SnO nanoribbons host edge-derived electronic states within the fundamental band gap. The nature of these states depends strongly on the edge composition: the O–O terminated ribbon [Fig. 4(d)] remains semiconducting, whereas the Sn–Sn and Sn–O terminations [Fig. 4(e) and (f)] exhibit metallic behaviour, with edge states intersecting the Fermi level. We analyze the contributions of edge and bulk atoms through PDOS calculations, where the blue and red curves represent the edge and bulk atom contributions, respectively. For all three edge configurations, the electronic states near the Fermi level are found to originate predominantly from the edge atoms, as clearly shown in the zoomed-in views of the DOS plots. This confirms the edge-localized nature of the states appearing around the Fermi energy. Such termination-dependent emergence of edge-localized bands is a general feature of oxide nanoribbons, in which reduced coordination and edge chemistry govern both the electronic structure and stability.^49–51^

Wavefunction isosurfaces plotted [see Fig. 4(g)–(i)] beneath each band structure confirm the strong spatial localization of these states at the ribbon boundaries. While the O–O termination yields localized in-gap states that do not contribute to conduction, Sn-containing edges support boundary states that extend along the ribbon direction, consistent with one-dimensional metallic channels. Furthermore, we plot the charge density distributions for the three types of edge nanoribbons in Fig. S3. The charge density analysis reveals that the Sn-terminated edge forms a delocalized charge channel along the Sn atoms, which gives rise to the metallic nature of the Sn-edge SnO nanoribbon.

To assess the relative stability of the three configurations, relative energies were computed. The relative energies of the O–O, Sn–Sn, and Sn–O terminated ribbons are −5.48 eV, −4.92 eV, and −5.17 eV per unit cell, respectively. The results show that the O–O terminated ribbon has the lowest formation energy, making it the most stable among the three. Although the Sn–Sn and Sn–O terminations exhibit metallic edge conduction, the O-rich configuration is energetically more stable.

The enhanced stability of the O–O terminated edge can be attributed to the effective saturation of dangling bonds and the resulting suppression of localized high-energy states at the boundary. Such a strong correlation between defect passivation, local coordination, and electronic transport is a recurring theme in oxide semiconductors, where the balance between structural stability and carrier delocalization critically governs device performance.^5,12^ In contrast, Sn-rich edges inherently retain partially filled Sn-derived orbitals, which promote metallic edge conduction but incur a higher energetic cost. This trade-off between structural stability and electronic conductivity highlights a general design principle for square-lattice oxide nanoribbons: oxygen-rich terminations favor thermodynamic stability, whereas cation-rich edges enable low-dimensional metallic transport.

These results reveal a clear trade-off between structural stability and electronic conductivity in SnO nanoribbons. Oxygen-rich edge terminations effectively saturate dangling bonds and thus minimize high-energy localized states, leading to enhanced thermodynamic stability. In contrast, tin-rich edges inherently retain partially filled Sn-derived orbitals, which promote metallic edge conduction at the cost of higher formation energies. Such a balance between energetic stability and low-dimensional conductivity reflects a general design principle in low-dimensional oxide systems, where local coordination and defect chemistry play decisive roles in determining electronic functionality.^52^ Accordingly, chiral edge engineering combined with controlled edge passivation provides a viable route for tailoring the electronic properties of SnO nanoribbons.

## 4. Conclusion

In conclusion, we have systematically investigated the electronic, magnetic, and optical properties of monolayer SnO through transition-metal doping and edge engineering using first-principles calculations. Substitutional Co doping introduces strongly spin-polarized impurity states near the Fermi level, giving rise to an apparent half-metallic behavior within the DFT-PBE approximation. However, upon inclusion of on-site Coulomb interaction, the half-metallic character disappears due to correlation-driven splitting of the localized impurity states. These findings indicate that Co-doped SnO exhibits dilute localized magnetism, highlighting its potential for oxide-based spintronic applications.

We further demonstrated that SnO nanoribbons host robust edge-localized electronic states that persist across different edge terminations and passivation schemes, confirming their intrinsic origin. In addition, these localized edge states are largely independent of the ribbon width. Hydrogen passivation stabilizes the edge structures without eliminating the edge states, providing a realistic platform for probing edge-dominated transport in oxide nanostructures. For chiral nanoribbons oriented along low-symmetry directions, a clear trade-off emerges between thermodynamic stability and electronic conductivity: oxygen-rich edges favor structural stability, whereas tin-rich edges enable metallic one-dimensional conduction channels.

The present results also suggest that the proposed strategy of combining transition-metal doping and edge engineering may be extended to other oxide semiconductors. However, the emergence of localized flat-band states and edge-dependent metallicity in SnO is strongly influenced by its unique lone-pair-driven electronic structure arising from Sn 5s-O 2p hybridization, which distinguishes it from oxide systems such as SnO_2_ and In_2_O_3_. Therefore, while the general design principles may be broadly applicable, the resulting electronic and magnetic behaviors are expected to depend sensitively on the underlying orbital characteristics of the host oxide material.

These findings establish general design principles for low-dimensional oxide semiconductors, where electronic functionality can be tuned through a delicate interplay of dopant chemistry, local coordination, and edge composition. The present work thus positions monolayer SnO as a versatile platform for exploring multifunctional nanoelectronic and spintronic phenomena in oxide-based two-dimensional materials.

## Conflicts of interest

There are no conflicts to declare.

## Supplementary Material

NA-008-D6NA00140H-s001

## Data Availability

All relevant data supporting the findings of this study are included in the article and its supplementary information (SI). Additional data or computational files are available from the corresponding author upon reasonable request. Supplementary information: additional computational details, electronic structures, spin-density distributions, and supporting data. See DOI: https://doi.org/10.1039/d6na00140h.
