# Supplementary material for: Dilute magnetism and edge-state engineering in monolayer SnO
Source: Nanoscale Adv. 2026 Jun 2;8(14):4101–8. doi: 10.1039/d6na00140h (PMC13274704; doi:10.1039/d6na00140h)
Supplement: NA-008-D6NA00140H-s001 [file NA-008-D6NA00140H-s001.pdf]

Supplementary Information (SI) for Nanoscale Advances.
This journal is © The Royal Society of Chemistry 2026

Supplemental Material for "Dilute Magnetism and Edge-State Engineering in Monolayer SnO"

Yuya Fukuta,[1] Souren Adhikary,[1] Kazuhito Tsukagoshi,[2] and Katsunori Wakabayashi[1,2,3]

[1] *Department of Nanotechnology for Sustainable Energy, School of Science and Technology, Kwansei Gakuin University, Gakuen-Uegahara 1, Sanda 669-1330, Japan*
[2] *Research Center for Materials Nanoarchitectonics (MANA), National Institute for Materials Science (NIMS), Namiki 1-1, Tsukuba 305-0044, Japan*
[3] *Center for Spintronics Research Network (CSRN), Osaka University, Toyonaka 560-8531, Japan*

I. ELECTRONIC BAND STRUCTURE OF S*n*O@C*o* IN PRESENCE OF ON-SITE COULOMB INTERACTION

Here, we present the electronic band structures of the Co-doped SnO monolayer in the presence of on-site Coulomb interaction (U). The U parameter is applied to the Co *d*-orbitals, and its value is varied from 1 to 3 eV [Figs. S1(b)–S1(d)]. For comparison, the electronic band structure without U is also shown in Fig. S1(a). The results show that the half-metallic character disappears upon inclusion of U=1 eV due to correlation-induced splitting of the impurity states. However, for higher values of U (U=2 and 3 eV), the overall band dispersion remains qualitatively unchanged. These results indicate that the flat-band nature and localized character of the impurity states are robust against variations in the on-site Coulomb interaction.

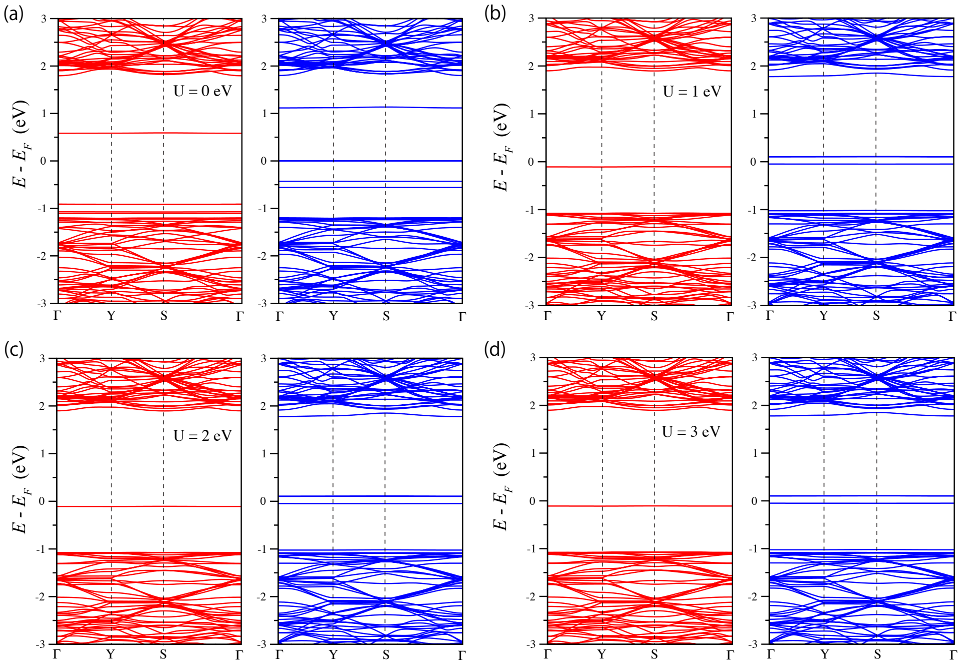

FIG. S1. Electronic band structure of Co doped SnO monolayer in presence of U.

II. ELECTRONIC PROPERTIES OF SnO NANORIBBON WITH DIFFERENT WIDTH

We consider SnO nanoribbons with different widths compared to the ribbon shown in Fig.3 of the main text. In Fig.S2, we present the electronic band structures and the wavefunction distributions corresponding to the degenerate bands near the Fermi level, marked by the red arrows, for SnO nanoribbons of different widths. The wavefunction analysis reveals that the states near the Fermi level primarily originate from the edges of the corresponding ribbons. Furthermore, although the band gap varies with ribbon width, the overall nature of the band dispersion remains unchanged. These results confirm that the edge-localized states are robust against variations in ribbon width.

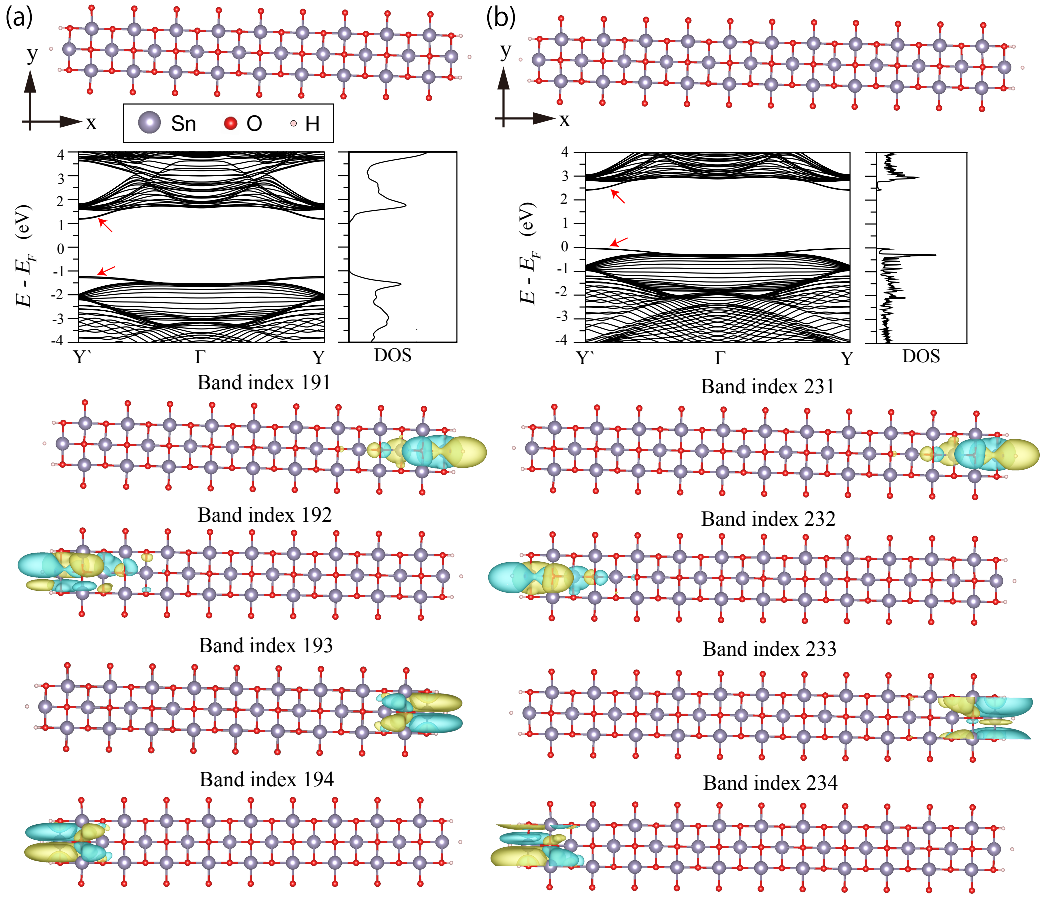

FIG. S2. Electronic band structure and wavefunction of SnO nanoriobbon with different width. The isosurface value is set to 0.001 $e/\text{Å}^3$.

III. CHARGE DENSITY PLOT OF CHIRAL EDGE SnO NANORIBBON

Here, we present the charge density distributions of chiral-edge SnO nanoribbons with three different edge terminations. For the O–O and Sn–O edge configurations, the charge density is primarily localized along the edge oxygen atoms. In contrast, the Sn-dominated edge exhibits delocalized charge distribution along the Sn atoms. This charge delocalization gives rise to the metallic character observed in the Sn-dominated chiral SnO nanoribbon.

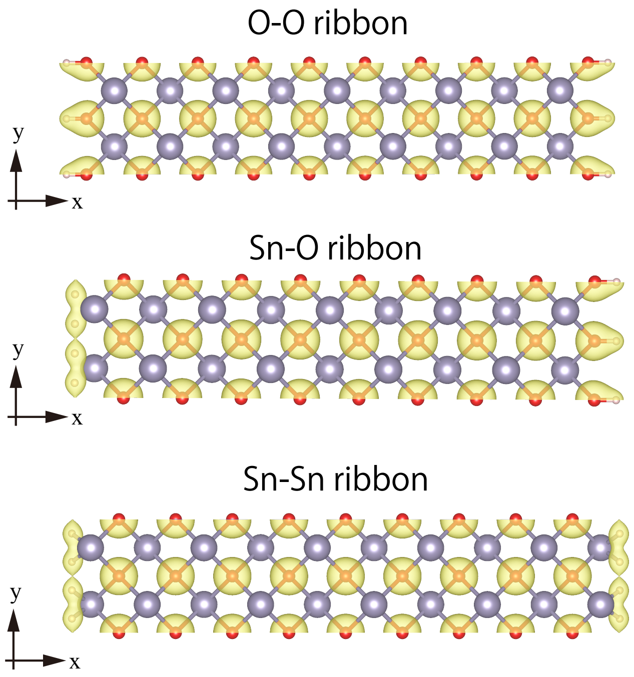

FIG. S3. Charge density plot of chiral edge SnO nanoribbon. The isosurface value is set to 0.001 $e/\text{Å}^3$.

[1] P. Hohenberg and W. Kohn, Inhomogeneous electron gas, Phys. Rev. **136**, B864 (1964).
[2] G. Kresse and J. Hafner, Ab initio molecular dynamics for liquid metals, Phys. Rev. B **47**, 558 (1993).
[3] P. E. Blöchl, Projector augmented-wave method, Phys. Rev. B **50**, 17953 (1994).
[4] J. P. Perdew, K. Burke, and M. Ernzerhof, Generalized gradient approximation made simple, Phys. Rev. Lett. **77**, 3865 (1996).
[5] S. Grimme, J. Antony, S. Ehrlich, and H. Krieg, A consistent and accurate ab initio parametrization of density functional dispersion correction (DFT-D) for the 94 elements H-Pu, J. Chem. Phys. **132** (2010).
[6] H. J. Monkhorst and J. D. Pack, Special points for brillouin-zone integrations, Phys. Rev. B **13**, 5188 (1976).
[7] G. Kresse and D. Joubert, From ultrasoft pseudopotentials to the projector augmented-wave method, Phys. Rev. B **59**, 1758 (1999).
[8] G. Pizzi, V. Vitale, R. Arita, S. Blügel, F. Freimuth, G. Géranton, M. Gibertini, D. Gresch, C. Johnson, T. Koretsune, *et al.*, Wannier90 as a community code: new features and applications, J. Phys. Conden. Matt. **32**, 165902 (2020).
[9] A. A. Mostofi, J. R. Yates, Y.-S. Lee, I. Souza, D. Vanderbilt, and N. Marzari, Wannier90: A tool for obtaining maximally-localised wannier functions, Comp. Phys. Commun. **178**, 685 (2008).
[10] J. R. Yates, X. Wang, D. Vanderbilt, and I. Souza, Spectral and Fermi surface properties from Wannier interpolation, Phys. Rev. B **75**, 195121 (2007).
[11] G. Dvoryankina and Z. Pinsker, A study of thin layers of phases in the Ni-Te system. A study of the beta-phase of NiTe, Sov. Phys. Crystallogr. **8**, 448 (1964).
[12] F. Zheng, X.-B. Li, P. Tan, Y. Lin, L. Xiong, X. Chen, and J. Feng, Emergent superconductivity in two-dimensional $NiTe_2$ crystals, Phys. Rev. B **101**, 100505 (2020).
